# Clinical and microbiological features of obstructive cholangitis with bloodstream infection caused by *Pandoraea apista* identified by MALDI-TOF mass spectrometry and ribosomal RNA sequencing in a cancer patient

**DOI:** 10.1186/s12879-022-07514-z

**Published:** 2022-06-07

**Authors:** Naoya Itoh, Nana Akazawa, Yuichi Ishibana, Toshiki Masuishi, Akinobu Nakata, Hiromi Murakami

**Affiliations:** 1 Division of Infectious Diseases, Aichi Cancer Center Hospital, 1-1 Kanokoden, Chikusa-ku, Nagoya, Aichi 464-8681 Japan; 2 Collaborative Chairs Emerging and Reemerging Infectious Diseases, National Center for Global Health and Medicine, Graduate School of Medicine, Tohoku University, 2-1 Seiryo-machi, Aoba-ku, Sendai, Miyagi 980-8575 Japan; 3 Department of Clinical Oncology, Aichi Cancer Center Hospital, 1-1 Kanokoden, Chikusa-ku, Nagoya, Aichi 464-8681 Japan

**Keywords:** *Pandoraea apista*, Matrix-assisted laser desorption/ionization time-of-flight mass spectrometry, Ribosomal RNA sequencing, Cholangitis, Bloodstream infection, Bacteremia

## Abstract

**Background:**

*Pandoraea* species are multidrug-resistant glucose-nonfermenting gram-negative bacilli that are usually isolated from patients with cystic fibrosis (CF) and from water and soil. Reports of diseases, including bloodstream infections, caused by *Pandoraea* spp. in non-CF patients are rare, and the clinical and microbiological characteristics are unclear. The identification of *Pandorea* spp. is limited by conventional microbiological methods and may be misidentified as other species owing to overlapping biochemical profiles. Here, we report the first case of obstructive cholangitis with bacteremia caused by *Pandoraea apista* in a patient with advanced colorectal cancer.

A 61-year-old man with advanced colorectal cancer who underwent right nephrectomy for renal cell carcinoma 4 years earlier with well-controlled diabetes mellitus was admitted to our hospital with fever for 2 days. The last chemotherapy (regorafenib) was administered approximately 3 weeks ago, and an endoscopic ultrasound-guided hepaticogastrostomy was performed 2 weeks ago under hospitalization for obstructive jaundice. Two days prior, he presented with fever with chills. He was treated with piperacillin-tazobactam for obstructive cholangitis and showed improvement but subsequently presented with exacerbation. Bacterial isolates from the blood and bile samples were identified as *P. apista* using matrix-assisted laser desorption/ionization time-of-flight mass spectrometry (MALDI-TOF MS) and 16S ribosomal RNA sequencing. Based on the susceptibility results of the isolates, he was successfully treated with oral trimethoprim-sulfamethoxazole 160 mg/800 mg/day for 14 days for *P. apista* infection.

**Conclusions:**

*Pandoraea* species are often misidentified. Therefore, multiple approaches should be used to identify them, and decisions regarding antimicrobial treatment should be based on actual in vitro susceptibility. Only seven cases of *Pandoraea* spp. bloodstream infections have been reported, and we report the first case of cholangitis with bacteremia.

## Background

The *Pandoraea* genus was first described by Coenye et al. in Belgium in 2000 [1]. *Pandoraea* species are multidrug-resistant, non-fermenting, gram-negative bacilli, usually isolated from patients with cystic fibrosis (CF) and the environment, such as water and soil [1, 2]. Although reports of *Pandoraea* spp. isolated from humans are scarce, the geographic regions in which these cases are described are consistent with the epidemiology of patients with CF in Europe, the United States, and Australia [3, 4]. Additionally, there is limited evidence of infections caused by the emerging opportunistic pathogen *Pandoraea* spp. in patients without CF [3]. Conventional microbiological methods for the identification of bacterial species have limitations and may not be very accurate, possibly leading to misidentification of *Pandoraea* as other species, such as *Ralstonia*, *Stenotrophomonas*, or *Burkholderia* species, owing to overlapping biochemical profiles [5]. Thus, the prevalence of *Pandoraea* spp. may have been underestimated. The pathogenicity of *Pandoraea* spp. also remains unknown, and all isolates are multidrug-resistant. Therefore, understanding and accurately identifying these organisms are important.

In this study, we report the first case of cholangitis associated with bacteremia caused by *P. apista* identified by matrix-assisted laser desorption/ionization-time-of-flight mass spectrometry (MALDI-TOF MS) and 16S ribosomal RNA (16S rRNA) sequencing in a patient with advanced colorectal cancer. Furthermore, this is the first report of a human infection caused by *Pandoraea* spp. in Japan.

## Case presentation

**Fig. 1:**
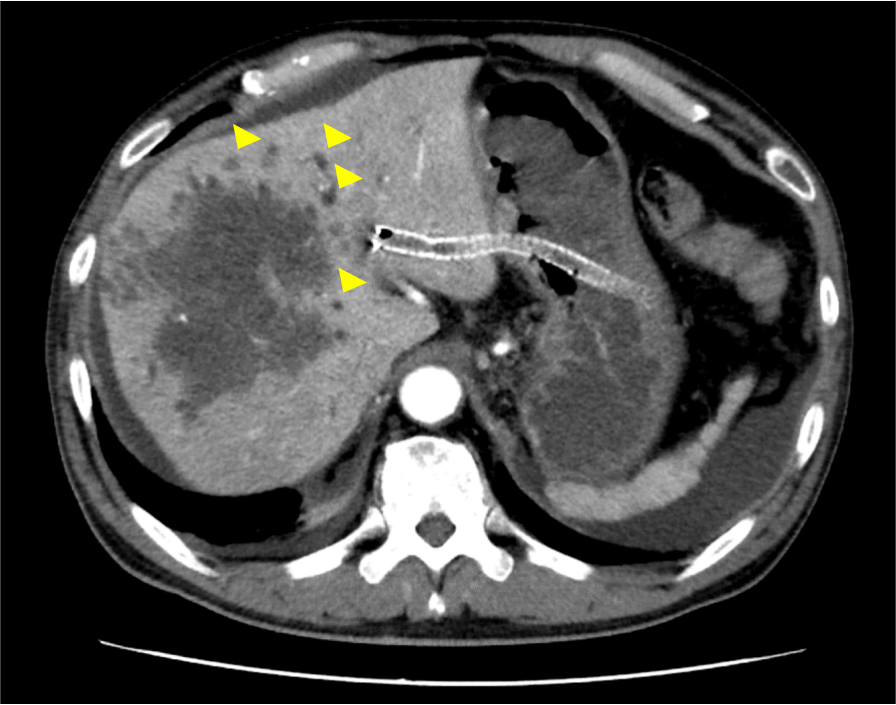
Computed tomography showing hepatic metastases and dilated peripheral bile ducts (yellow triangle) and hepaticogastrostomy stenting

**Fig. 2:**
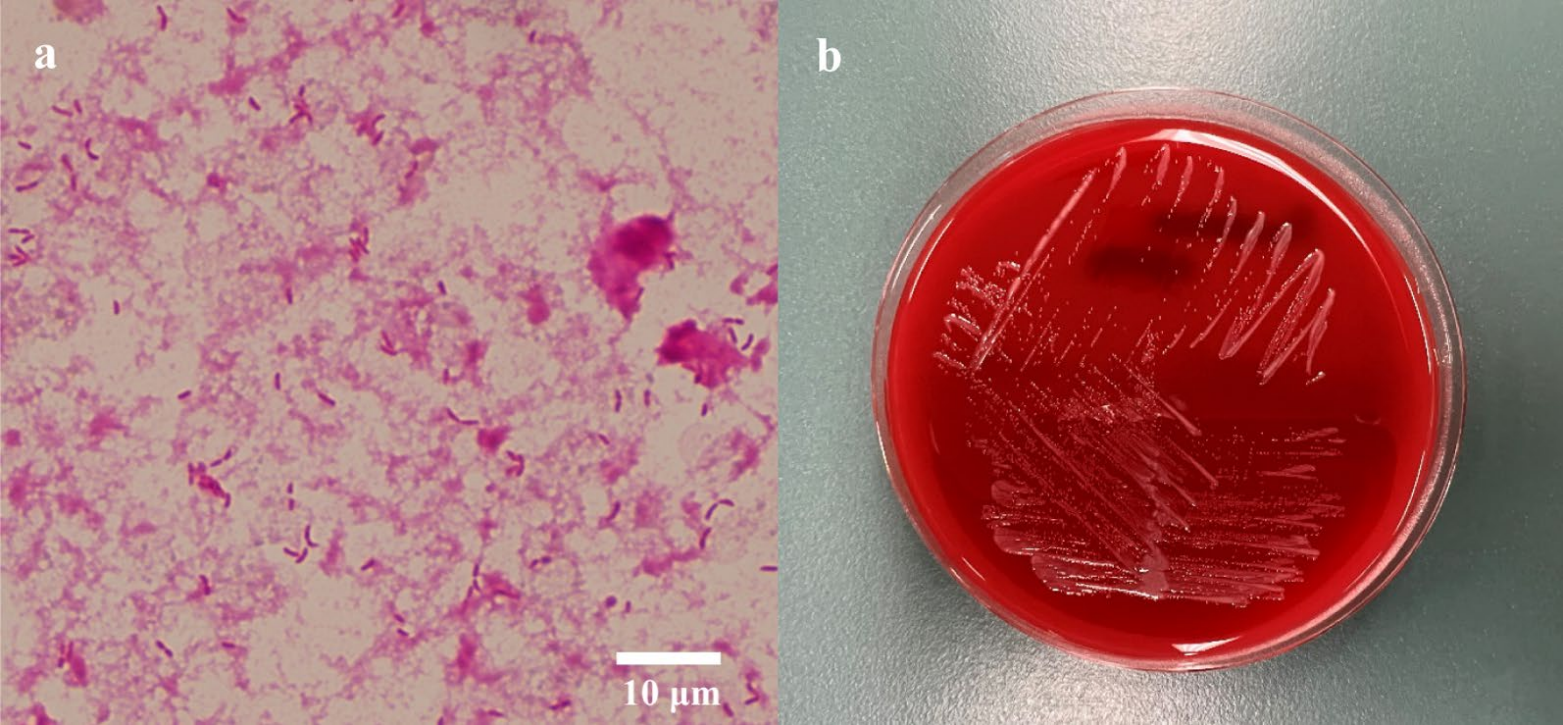
Blood culture media revealing small gram-negative bacilli staining, Gram staining, 1000 × (**a**). White colonies of *Pandoraea apista* after 24 h on sheep blood agar plate (**b**)

A 61-year-old Japanese man with advanced colorectal adenocarcinoma following right nephrectomy for clear cell renal cell carcinoma 4 years earlier and well-controlled type 2 diabetes presented with fever accompanied by chills (Day 0). Renal cell carcinoma presented with liver metastasis nearly a year after surgery, and he was treated with sunitinib and nivolumab for approximately a year. He was diagnosed as having colorectal cancer with liver metastasis approximately 2.5 years ago. He currently had stage IV colorectal cancer with multiple metastases to the liver, lung, and hilar lymph nodes. He was treated with bevacizumab plus infusional 5-fluorouracil/leucovorin plus oxaliplatin (FOLFOX), bevacizumab plus infusional 5-fluorouracil/leucovorin plus irinotecan (FOLFIRI), bevacizumab plus trifluridine/tipiracil (FTD/TPI), and regorafenib for approximately 2.5 years. The last chemotherapy session (regorafenib) was performed approximately 3 weeks ago. Two weeks earlier, he was hospitalized and underwent endoscopic ultrasound-guided hepaticogastrostomy (EUS-HGS) for obstructive jaundice. He was discharged 4 days earlier. Two days prior, he had chills and developed a fever of 39℃. Upon examination, he appeared well, and the chills were gone. His temperature was 38.1℃, heart rate was regular at 105 bpm, blood pressure was 127/81 mmHg, and oxygen saturation was 96% in room air. His abdomen was flat, soft, and non-tender. Other physical examination results were unremarkable. Laboratory investigations revealed a white blood cell count of 18,370/mL (neutrophil count: 14,696/mL), normocytic anemia (hemoglobin level: 11.5 g/dL), C-reactive protein at 33.50 mg/dL (normal < 0.30 mg/dL), HbA1c levels of 6.5% (normal 4.6–6.2%), persistently elevated serum hepatic markers (gamma-glutamyltransferase: 297 U/L [normal < 30 U/L], alkaline phosphatase: 1289 U/L [normal 115–359 U/L], total bilirubin: 8.2 mg/dL [normal 0.3–1.2 mg/dL], and direct bilirubin: 5.5 mg/dL [normal 0.0–0.2 mg/dL]), elevated serum renal markers (blood urea nitrogen: 36.2 mg/dL [normal 8.0–20.0 mg/dL]), creatinine: 1.63 [normal 0.65–1.07 mg/dL], and elevated tumor markers (carbohydrate antigen 19–9: 3971 U/mL [normal < 37.0 U/mL] and carcinoembryonic antigen of 1486.5 ng/mL [normal < 5.0 ng/mL]). Abdominal computed tomography (CT) revealed hepatic metastases, dilated peripheral bile ducts, and HGS stenting (Fig. 1). As an empirical antimicrobial therapy for obstructive cholangitis, intravenous piperacillin-tazobactam (PIPC/TAZ) at a dose of 9 g/day was administered. The fever had resolved by Day 2 without using antipyretics. Blood cultures collected upon admission (Day 0) yielded negative results. Fever and elevated bilirubin levels were observed on Day 8. A plastic stent was inserted into the posterior hepatic area during endoscopic retrograde cholangiopancreatography (ERCP) on Day 10 for relieving the bile duct obstruction, and bile culture was performed. The blood cultures collected on Day 8 and Day 12 revealed negative findings. The fever continued, and blood culture was obtained again on Day 15. Gram-negative rods were observed after 24 h and 25 min (Fig. 2a).

Positive bottles were subcultured on sheep blood agar plates (Nissui Pharmaceutical Co., Ltd., Tokyo, Japan). After 24 h of aerobic incubation at 35 °C, white colonies were observed (Fig. 2b). Subsequently, the blood sample results identified the bacterial culture as *Pandoraea* spp. with 99% confidence using VITEK 2 ver. 9.02 (SYSMEX bioMérieux Co., Ltd., Tokyo, Japan). The culture results of the bile specimens also identified *Pandoraea* spp. using VITEK 2 (95% confidence). Bile and blood culture samples were analyzed by MALDI-TOF MS using VITEK MS ver. 4.7.1 (bioMérieux, Tokyo, Japan), which also identified *P. apista* with a 99.9% confidence value. 16S rRNA sequencing was performed on strains identified as *P. apista* acquired from the blood culture samples. After performing a BLAST Basic Local Alignment Search Tool search of the 16S rRNA sequence of the isolated strain, a 100% homology (1455/1455 bp) with the standard *P. apista* strain TF80G25 (GenBank Accession No.: CP011279.2) was obtained. Antimicrobial susceptibility tests were performed according to the methodology recommended by the Clinical and Laboratory Standards Institute document M100-S25 (2015) for other non-Enterobacteriaceae isolates as there are no interpretive criteria for this strain. Both *P. apista* isolates from the bile and blood cultures were found to be susceptible to trimethoprim-sulfamethoxazole (Table 1).

**Table 1:** Antibiotic susceptibility of isolated *Pandoraea apista*

	MIC (μg/mL) of bile sample	Susceptibility of bile sample	MIC (μg/mL) of blood sample	Susceptibility of blood sample
Piperacillin	≥ 128	R	≥ 128	R
Piperacillin-tazobactam	≥ 128	R	≥ 128	R
Ceftazidime	≥ 64	R	≥ 64	R
Cefepime	16	I	32	R
Ceftriaxone	32	I	≥ 64	R
Imipenem	≥ 16	R	≥ 16	R
Meropenem	≥ 16	R	≥ 16	R
Gentamicin	8	I	≥ 16	R
Amikacin	16	S	≥ 64	R
Minocycline	8	I	8	I
Ciprofloxacin	≥ 4	R	≥ 4	R
Levofloxacin	4	I	4	I
Trimethoprim-sulfamethoxazole	≤ 20	S	≤ 20	S

*MIC* minimal inhibitory concentration, *S* susceptible, *I* intermediate, *R* resistant

Careful physical examination revealed no obvious disseminated lesions suspected of septic embolization. On Day 16, the fever resolved without any change in the antimicrobial therapy with PIPC/TAZ. On Day 18, based on the patient’s bile and blood culture results and his request for discharge, the medication was switched to oral antimicrobials. Considering the success of our treatment with PIPC/TAZ, we switched to oral amoxycillin-clavulanic acid (1000 mg/250 mg/day) and levofloxacin (250 mg/day) for 14 days. We also prescribed oral trimethoprim-sulfamethoxazole (160 mg/800 mg/day) for 14 days as treatment for his *P. apista* infection. He was discharged on Day 17, and there was no recurrence of fever at the 1-month follow-up.

## Discussion and conclusion

Bloodstream infections caused by *Pandoraeae* spp. are extremely rare, and there have been no reports of its association with cholangitis. To our knowledge, this study reports the first case of obstructive cholangitis with *P. apista* bacteremia. The findings of this research will be beneficial to clinicians because it provides additional information about the lesser known *Pandoraea* species.

The *Pandoraea* genus was first described by Coenye et al. in 2000 [1]. This genus is a novel emerging pathogen group belonging to the β-subclass of Proteobacteria, with the genera *Burkholderia* and *Ralstonia* as its closest neighbors [1]. The genus *Pandoraea* comprises aerobes or facultative anaerobes, non-sporing, non–lactose fermenters, o-nitrophenyl-β-D-galactopyranoside-negative, and gram-negative bacilli with polar flagella [2]. Currently, there are five known *Pandoraea* species isolated from human clinical specimens (*P. apista*, *P. pulmonicola*, *P. pnomenusa*, *P. sputorum*, and *P. norimbergensis*), five non-clinical species (*P. thiooxydans*, *P. oxalativorans*, *P. faecigallinarum*, *P. vervacti*, and *P. terrae*), and at least four unnamed genomospecies [3].

Most cases of *Pandoraea* infection consistent with the epidemiology of CF have occurred in Europe, America, and Australia and are rarely reported in Asia [3, 4, 6]. In Japan, there have been no reports of human infection or colonization caused by *Pandoraea* spp., except for the present case. The scarcity of reports, besides the factors of CF epidemiology, may imply that the identification of *Pandoraea* spp. might be underestimated owing to the difficulty of routine diagnostic tests. Applying only commonly used tests such as conventional phenotypic methods and the VITEK 2 automated microbial system, microbiology laboratories often misidentify this pathogen as *Ralstonia*, *Stenotrophomonas*, or *Burkholderia* spp. [7–9]. Additionally, 16S rRNA analysis and gyrB gene sequences are reliable but also have limitations for the identification of *Pandoraea* spp. [9, 10]. Recently, it has been reported that MALDI-TOF MS shows good results in the identification of many bacterial species [3, 5, 6]. MALDI-TOF MS has demonstrated its accuracy and usefulness as a technique for the rapid identification of bacteria, but one of its limitations is the limited reference dataset for microorganisms that are less frequently isolated from clinical specimens [11]. As each test has its own limitations, multiple approaches are ideal for accurate microbial identification. In the present case, the combination of 16S rRNA sequencing and MALDI-TOF MS enabled us to identify all the microorganisms isolated from the blood and bile samples as *P. apista*. Surprisingly, in VITEK 2, the organism was also identified as *Pandoraea* spp. Updating the database of all characterization instruments, including automated microbial identification systems and MALDI-TOF MS, may be important for accurate identification of less studied bacterial species. Furthermore, updating the database suggests that accurate diagnoses can be made without using advanced and expensive molecular diagnostic techniques, such as 16S rRNA sequencing, and with less cost and time using routine practices with common instruments, such as VITEK 2 and MALDI-TOF MS, in a normal laboratory. To the best of our knowledge, only eight case reports of bloodstream infections caused by *Pandoraea* spp. have been published (Table 2) [6, 12–17].

**Table 2:** Summary of published cases of blood stream infection due to *Pandoraea* species

Author, country, year, reference	Age (years), sex	Strains	Symptoms	Underlying condition	Diagnostic method	Susceptibility MIC (μg/mL)	Other bacteria isolated	Treatment for *Pandoraea*	Outcome
Stryjewski et al., 2003 (USA) [12]	30, M	*P. pnomenusa*	Fever, hypotension	Pneumonia, pulmonary sarcoidosis, lung transplantation	PCR, gyrB gene sequencing	Zone diameter: IPM 33 mm; MEPM 6 mm	None	IPM, until dead	Died
Johnson et al., 2004 (USA) [13]	16, M	*P. apista*	Fever	CLABSI, cystic fibrosis	16S rRNA sequencing	ND	*Candida albicans*	CVC removal, IPM and CTRX, 14 days	Survived
Falces-Romero et al., 2016 (Spain) [14]	10 months, F	*P. pnomenusa*	Fever, shaking chill	CLABSI, Leukemia	MALDI-TOF MS	Susceptible for MINO and IPM (Details unknown)	None	CVC removal, IPM, 10 days	Survived
Xiao et al., 2019 (China) [15]	43, M	*P. sputorum*	Elevated inflammatory markers	Hepatocellular carcinoma, liver transplantation	MALDI-TOF MS, 16S rRNA sequencing	AMK 32; AZT > 64; CFPM > 64; CAZ > 64; CPFX ≤ 0.5; CTRX ≤ 0.5; GM > 16; IPM ≤ 0.5; LVFX ≤ 1; MEPM 16; MINO ≤ 1; PIPC ≤ 16; PIPC/TAZ ≤ 8/4; ST ≤ 38/2	None	IPM and CTRX/TAZ	Survived
Gawalkar et al., 2020 (India) [16]	42, M	*P. pnomenusa*	Fever, dyspnea	IE, prosthetic aortic valve replacement	ND	Susceptible for LVFX, MINO, ST (Details unknown)	none	LVFX	Died
Bodendoerfer et al., 2021 (Switzerland) [17]	37, M	*P. pnomenusa*	Fever	CLABSI, prosthetic valve endocarditis, IDU	Whole-genome sequencing	Strain 1: CAZ 24; CPFX 0.5; IPM 1.5; MEPM ≥ 32; MINO 0.38; PIPC 24; PIPC/TAZ 0.19; ST 0.64	none	CVC removal, PIPC/TAZ, ST	Survived
						Strain 2: CAZ 16; CPFX 0.5; IPM 1.5; MEPM ≥ 32; PIPC 8; PIPC/TAZ 0.047; ST 0.64			
Singh et al., 2021 (India) [6]	72, M	*P. apista*	Fever, dyspnea, gastric upset, diarrhea	COVID-19	MALDI-TOF MS	Susceptible for IPM, MINO, DOXY and ST; Resistant for CAZ, MEPM, PIPC/TAZ, colistin, AMK, CPZ/SBT (Details unknown)	none	IPM	Survived
Present case	61, M	*P. apista*	Fever	Colorectal cancer, renal cell carcinoma, DM	MALDI-TOF MS, 16S rRNA sequencing	See Table 1	none	ST	Survived

*M* male, *F* female, *MIC* minimum inhibitory concentration, *CLABSI* central line-associated bloodstream infection, *CVC* central venous catheter, *PCR* polymerase chain reaction, *rRNA* ribosomal RNA, *MALDI-TOF MS* matrix-assisted laser desorption/ionization-time-of-flight mass spectrometry, *AMK* amikacin, *AZT* aztreonam, *CFPM* cefepime, *CAZ* ceftazidime, *CPFX* ciprofloxacin, *CTRX* ceftriaxone, *GM* gentamycin, *IPM* imipenem, *LVFX* levofloxacin, *MEPM* meropenem, *MINO* minocycline, *PIPC* piperacillin, *PIPC/TAZ* piperacillin/tazobactam, *ST* sulfamethoxazole trimethoprim, *IDU* injecting drug user, *DOXY* doxycycline, *CPZ/SBT* cefoperazone-sulbactam, *COVID-19* coronavirus disease 2019, *DM* diabetes, *ND* no data, *IE* infective endocarditis

There was only one case of bloodstream infection caused by *Pandoraea* spp. reported in a patient with CF [13] and six cases, including the present case, were all of non-CF patients [6, 12, 14–17]. In one report of a patient with CF, catheter-related bloodstream infection or pneumonia was considered the cause of the bloodstream infection [13]. Among the non-CF patients, five of the six had some type of immunodeficiency, including solid tumors and hematological malignancies. One patient had an organ transplantation [12], one contracted coronavirus disease 2019 (COVID-19) [6], and one had chronic rheumatic valve disease and underwent prosthetic aortic valve replacement [17]. Based on these findings, it is reasonable to consider that *Pandoraea* spp. can be an opportunistic pathogen in non-CF patients. The potential source of the bacteria in our case was considered to be attributed to the contamination of the bacteria from the environment during the EUS-HGS procedure, which resulted in cholangitis with bacteremia. Reports of bloodstream infections of *Pandoraea* spp. could increase in the future since the number of immunocompromised patients increases with advances in cancer treatment.

In our case, the patient was successfully treated with intravenous PIPC/TAZ and oral trimethoprim-sulfamethoxazole, amoxycillin-clavulanic acid, and levofloxacin. *P. apista* bacteremia developed under PIPC/TAZ treatment, and only *P. apista* was isolated from the bile. The susceptibility to PIPC/TAZ and levofloxacin for *P. apista* was resistant and intermediate, respectively. However, since other microorganisms that could potentially be effective for PIPC/TAZ were not identified in the culture, oral amoxycillin-clavulanic acid and levofloxacin were administered in addition to trimethoprim-sulfamethoxazole. Although he requested oral medication instead of intravenous treatment, the bioavailability of oral trimethoprim-sulfamethoxazole is nearly 100%, which is already considered acceptable as a treatment method [18]. Oral amoxycillin-clavulanic acid and levofloxacin are also favorable at a bioavailability of 60 ± 23% and > 95%, respectively [18]. According to reports, the susceptibility of *Pandoraea* spp. to trimethoprim-sulfamethoxazole is often preserved compared to that of other antimicrobial agents [15, 17]. To date, no breakpoints for antimicrobial susceptibility testing of *Pandoraea* spp. have been proposed; thus, the optimal treatment remains unknown. *Pandoraea* spp. are also known to be generally resistant to most antimicrobial agents, but their susceptibility to different antibiotics still varies, including piperacillin, PIPC/TAZ, aminoglycosides, and fluoroquinolones [3]. This organism often shows a unique pattern of resistance to both carbapenems and meropenem and a susceptibility to imipenem [3]. *Pandoraea* spp. has intrinsic carbapenem-hydrolyzing oxacillinases, which are responsible for carbapenem resistance [19, 20]. In addition to oxacillinase, they were also reported to have an efflux pump mechanism that contributes to their complicated multidrug resistance ability [3]. In the present case, *P. apista* was resistant to both meropenem and imipenem. This species has a unique antibiotic resistance pattern and can cause serious diseases, such as bloodstream infections, which require careful attention, especially when the pathogen cannot be clearly distinguished from other species. Therefore, antimicrobial therapy should be based on in vitro susceptibility testing.

Our report is limited in that it is a case report, and more case reports and case series are warranted to further understand the *Pandoraea* genus as a scope for future research.

We report the first case of obstructive cholangitis associated with bacteremia caused by *P. apista*. Because *Pandoraea* spp. are often misidentified, multiple approaches should be used for accurate identification and treatment decisions should be based on actual in vitro susceptibility results.

## Data Availability

The data used and/or analyzed during the current study are available from the corresponding author upon reasonable request.

## Abbreviations

BLAST: Basic Local Alignment Search Tool
CF: Cystic fibrosis
CT: Computed tomography
ERCP: Endoscopic retrograde cholangiopancreatography
EUS-HGS: Endoscopic ultrasound-guided hepaticogastrostomy
FOLFIRI: Infusional 5-fluorouracil/leucovorin plus irinotecan
FOLFOX: Infusional 5-fluorouracil/leucovorin plus oxaliplatin
FTD/TPI: Trifluridine/tipiracil
MALDI-TOF MS: Matrix-assisted laser desorption/ionization time-of-flight mass spectrometry
PIPC/TAZ: Piperacillin-tazobactam
rRNA: Ribosomal RNA
